# Retraction: Lamin B2 promotes the progression of triple negative breast cancer via mediating cell proliferation and apoptosis

**DOI:** 10.1042/BSR-2020-3874_RET

**Published:** 2024-05-31

**Keywords:** breast cancers, cell proliferation, Lamin B2 (LMNB2), triple negative breast cancer (TNBC)

This article is being retracted from *Bioscience Reports* at the request of the Editor-in-Chief and the Editorial Board following receipt of a notification from a reader, alerting the Editorial Board to similarities present between data in this manuscript and those in other articles by other authors. Specifically, the following similarities were noted:

- The tumour images in Figure 6A share similarities with images found in Figure 4 from Chen & Zhong, 2019 (DOI: 10.1155/2019/6383685) and Figure 4 from Sun et al, 2020 (doi: 10.1155/2020/6403012)
- The second (shRNA) panel in Figure 6D shares similarities with images found in Figure 4C from Chen & Zhong, 2019 (DOI: 10.1155/2019/6383685)
- The first (control) panel from Figure 6D shares similarities with the shRNA panel in Figure 4A from Sun et al, 2020 (doi: 10.1155/2020/6403012) and the control panel from 5B from Sun et al, 2019 (https://doi.org/10.1186/s13578-019-0340-9), the control panel from Figure 5D from Yao et al, 2014 (DOI: 10.3892/ol.2019.10040) and the control panel from Figure 4C from Li et al. 2019 (DOI: 10.1155/2019/9751923)
- The second (shRNA) panel of Figure 6C shares similarities with the shRNA panel of Figure 5C from Yuan et al 2020 (DOI: 10.7150/jca.39760)
- The Western blots from Figure 4B share similarities between the western blots from Figure 4D from Gao et al. 2020 (doi: 10.21037/atm-20-1970)

The authors have been in contact with the journal and provided repeated experimental data, including raw western blots for the new data; however, they have stated that the tumour and stained-glass slices have been lost. Splice lines were noted in the new raw data provided, which the authors explained was due to re-ordering of the bands after inconsistencies during loading samples. They also stated that many of the authors of papers where similarities were noted are friends and/or colleagues and have suggested that data could have been mixed up between the labs through errors in communication or naming/storage conventions. The Editorial Board feel that aspects of these explanations lead to doubt over the validity of the data and the scientific practices utilised, therefore they stand by the decision to retract the article. The authors agree to the retraction.

